# Rapid Detection of Ascorbic Acid Based on a Dual-Electrode Sensor System Using a Powder Microelectrode Embedded with Carboxyl Multi-Walled Carbon Nanotubes

**DOI:** 10.3390/s17071549

**Published:** 2017-07-02

**Authors:** Bao-Shan He, Jun-Xia Zhang

**Affiliations:** School of Food Science and Technology, Henan University of Technology, Lianhua Road 100#, Zhengzhou 450001, China; zhangjunxia6163@126.com

**Keywords:** ascorbic acid, multi-walled carbon nanotubes, platinum wire microelectrode, powder microelectrode

## Abstract

In this paper, carboxyl groups were introduced by liquid oxidation methods onto multi-walled carbon nanotubes (MWCNTs) to improve the MWCNTs’ electrocatalytic properties. A platinum wire microelectrode (ME) was corroded using aqua regia and subsequently embedded with MWCNTs to achieve more active sites, producing a so-called powder microelectrode (PME). Compared with conventional MEs, the PME has a larger specific surface area and more active sites. When PME was used to detect ascorbic acid (AA), the AA oxidation potential shifted negatively and current peak was visibly increased. The calibration curve obtained for AA was in a range of 5.00 × 10^−6^~9.50 × 10^−4^ mol·L^−1^: *I_pa_*(μA) = 3.259 × 10^−2^ *+* 1.801 × 10^2^ *C* (mol·L^−1^) under the optimum testing conditions. Moreover, the detection and quantitation limits were confirmed at 4.89 × 10^−7^ mol·L^−1^ and 1.63 × 10^−7^ mol·L^−1^, respectively. When the fabricated PME was practically applied to detect AA, it was shown a recovery rate of 94~107% with relative standard deviation (RSD) <5%. The proposed strategy thus offers a promising, rapid, selective and low-cost approach to effective analysis of AA.

## 1. Introduction

Ascorbic acid (AA) is an acid compound containing six carbon atoms and hydroxy groups, which is considered a natural organic acid although AA has no carboxyl group [1]. AA is soluble in water and insoluble in organic solvents. As an enol hexose acid lactone, the hydrogens from AA’s enol type hydroxyls are easily oxidized. AA is stable in an acidic environment, but when exposed to oxygen, heat, light, and alkaline substances, especially metal ions, AA tends to oxidize. Due to this easy oxidation, AA, which exists widely in fruits and vegetables, is lost to different degrees during storage. Therefore, the change of AA content represents an important index to judge the storage resistance and freshness of fruits and vegetables. Simultaneously, AA can be applied in the food industry as an antioxidant owing to its strong reducibility. In the human body, AA plays an important physiological role by participating in hydroxyl reactions, and preventing cancer [2], scavenging free radicals, etc. As the human body does not produce AA, it is important to include adequate AA intake in the diet. Lack of AA in the human body can lead to gum bleeding, anemia, and even scurvy [3,4,5]. Excess ingestion of AA will cause calculi and weaken body immunity. As a consequence, it is of great significance to establish an efficient, rapid and accurate detecting method for AA.

At present, common detection methods for AA include titration [6,7], liquid chromatography [8,9,10,11], spectrophotometry [12,13,14,15,16], fluorometry [17,18], and electrochemical methods [19,20,21,22,23]. Among these, titration is rapid and simple, but susceptible to the correct detection of the titration end point, which can affect the accuracy of the test results. The performance of liquid chromatography methods for AA detection is efficient, rapid and sensitive, but the tedious sample preparation steps can induce the loss of AA because of its easy oxidation. Electrochemical methods to determine AA have attracted attention owing to their low cost, simple instrumentation and good selectivity. As an improvement on macroelectrodes, microelectrodes (MEs) were introduced into the field of electrochemical analysis because of their small time constants, large diffusion speed and other advantages. When it comes to practical applications, however, MEs’ sensitivity do not achieve the desired effect. Thus, how to effectively improve MEs’ sensitivity for AA detection is the priority in this article.

Multi-walled carbon nanotubes (MWCNTs) have small diameters, high surface energy and a lack of coordination poperties. The ends display bending effects caused by pentagon defects. MWCNTs can be used as an ideal electrode-modifying material owing to their excellent electrical performance and high mechanical strength [24,25,26]. MWCNTs can significantly enhance the catalytic activity of electrodes and reduce the oxidation potential of reactants [27,28,29,30]. Especially, compared with conventional MEs, the functional powder microelectrode (PME) has a much larger response surface [31]. Though the apparent areas of the two kinds of electrode are equal, PMEs’ real surface areas are hundreds of even thousands of times higher than those of conventional MEs [32,33,34]. As a consequence, the same reactions on PMEs show higher apparent exchange current densities and better reversibility.

In this paper, MWCNTs, functionalized with carboxyl groups by pretreatment with strong mixed acid, were embedded into MEs for fabricating PMEs. Then, a two-electrode electrochemical system was constructed to detect AA based on its direct oxidation on the electrode surface using the PME as bass electrode and a platinum plate electrode as reference electrode.

## 2. Experimental

### 2.1. Chemicals

Multi-walled carbon nanotubes (MWCNTs) was supplied by Beijing Gaoke Technology Material Co., Ltd. (Beijing, China) and pretreated according to the previous report [21]. AA was purchased from Tianjin Kermel Chemical Reagent Co., Ltd. (Tianjin, China). Concentrated hydrochloric acid, nitric acid, disodium hydrogen phosphate and sodium dihydrogen phosphate were obtained from Luoyang Chemical Reagent Factory (Luoyang, China). The phosphate buffered solution (PBS) was prepared by mixing appropriate amounts of sodium dihydrogen phosphate and disodium hydrogen phosphate. All other reagents were analytical grade.

### 2.2. Apparatus

Electrochemical workstation CHI660E: Shanghai Chen Hua Instrument Co. (Shanghai, China); ΜB-7 acidity meter: Beijing Sartorius Scientific Instruments Co., Ltd. (Beijing, China); AR124CN electronic analytical balance: Shanghai Ohaus Instrument Co., Ltd. (Shanghai, China); KQ-3200E ultrasonic cleaner: Kunshan Ultrasonic Instrument Co., Ltd. (Kunshan, China); SZ-93 automatic dual water distiller, Shanghai YaRong Biochemical Instrument Factory (Shanghai, China); FTIR spectrometer: NICOLET 6700 Fourier infrared spectrometer (Thermo Fisher Scientific Co., Ltd., Madison, WI, USA); HT7700 Transmission electron microscope (TEM): Hitachi High-Technologies Co. (Tokyo, Japan).

## 3. Dual-Electrode AA Sensor System

### 3.1. Construction of Sensor System

**Figure 1:**
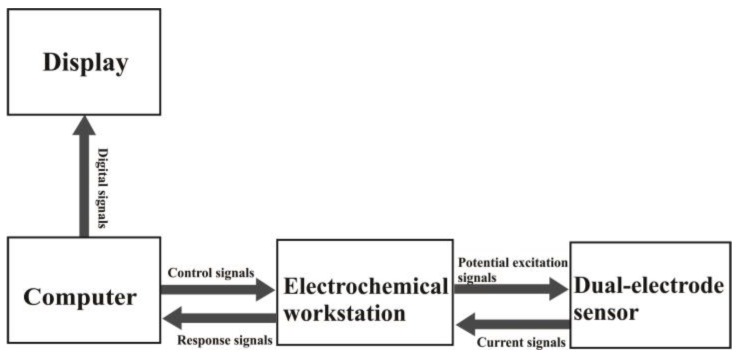
Schematic of the sensor system.

Figure 1 shows a schematic of the sensor system. An electrochemical workstation with a digital/analog (D/A), analog/digital (A/D) converter interface was taken as the center of the system. The potential pulse excitation signal was sent to the dual-electrode sensor system via the electrochemical workstation, so that the AA-sensing electrode was in real-time controlled at different potential states. The current response signals were dynamically acquired and displayed by the computer.

### 3.2. Fabrication of PME

**Figure 2:**
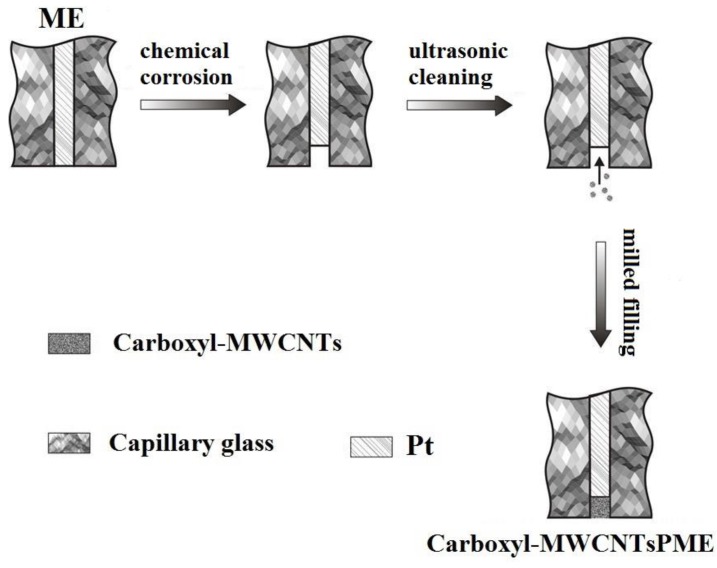
Fabrication of PME for AA detecting.

The fabrication process of the PME for AA detection is shown in Figure 2. Platinum wire of 50 μm diameter glued with copper wire was inserted into a pulled glass capillary to construct the ME. The tip of the pipet with platinum wire protruded out of the glass and was melted hermetically on an alcohol blowtorch. The junction between the glass micropipette and the copper wire was sealed with epoxy resin. Then the tip of platinum wire was burnished using metallographic sand paper and polished by alumina emulsion (1.0 μm, 0.3 μm, 0.05 μm) respectively. The platinum wire tip of the ME was corroded in boiling aqua regia to make microholes. Then carboxyl MWCNT powder on the smooth plate glass was placed vertically and repeatedly embedded into the microholes of the ME to form a PME.

### 3.3. Working Mechanism of the PME

**Figure 3:**
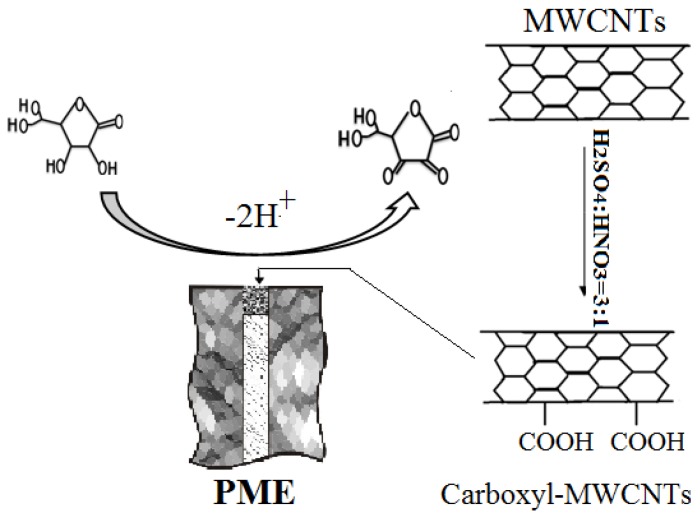
Working mechanism of PME for AA detection.

The working mechanism of the PME to detect the oxidation of AA in the presence of carboxyl MWCNTs is summarized in Figure 3. The as-fabricated PME can catalyze the two-electron electrocatalytic oxidation and hydrogen dissociation of AA to L-dehydroascorbic acid in the presence of oxygen in solution. The carboxyl MWCNTs acted as electron transfer mediators promoting the electron transfer between AA molecules and the matrix interface, thus enhancing the electrocatalytic oxidation of AA during electrochemical detection.

## 4. Results and Discussion

### 4.1. Characterization of the PME

#### 4.1.1. TEM of MWCNTs

**Figure 4:**
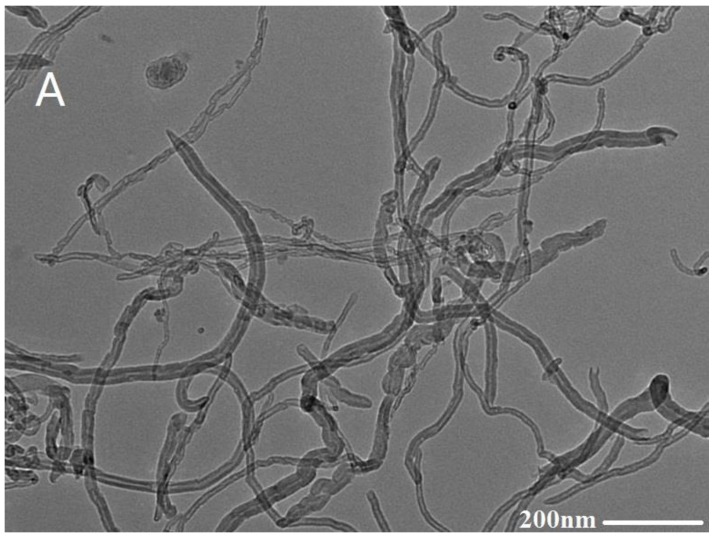

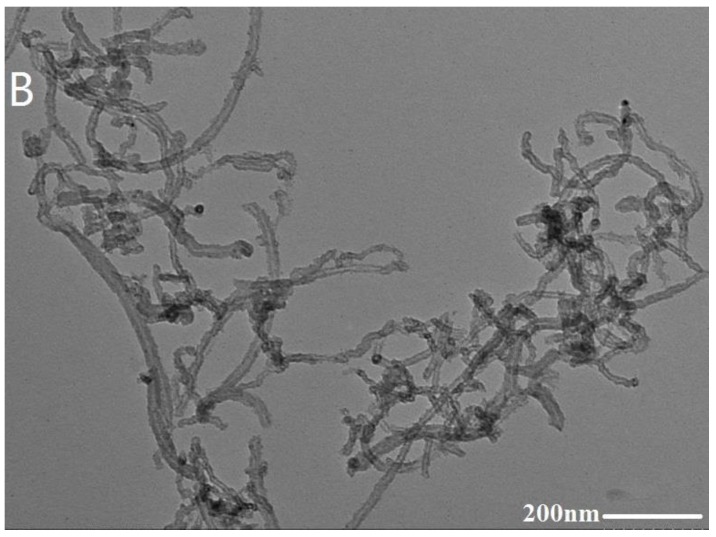
TEM images of MWCNTs (**A**) and carboxyl MWCNTs (**B**).

Transmission electron microscope (TEM) images of the multi-walled carbon nanotubes (A) and carboxyl multi-walled carbon nanotubes (B) are shown in Figure 4. Apparently, compared to MWCNTs, with lengths of 2600 nm~8000 nm, the length of carboxyl MWCNTs’ was decreased to 600 nm~5000 nm. The long tubes were thus split into shorter tubes during carboxyl treatment, hinting an increased number of open ends. The chain structures of carboxyl MWCNTs are ranked in an array of lines. The surface roughness of the carboxyl multi-walled carbon nanotubes chain increases. This indicates that the carboxyl MWCNTs have a larger specific surface area and more active sites than untreated MWCNTs, which is consistent with a previous report [21].

#### 4.1.2. FT-IR of MWCNTs

**Figure 5:**
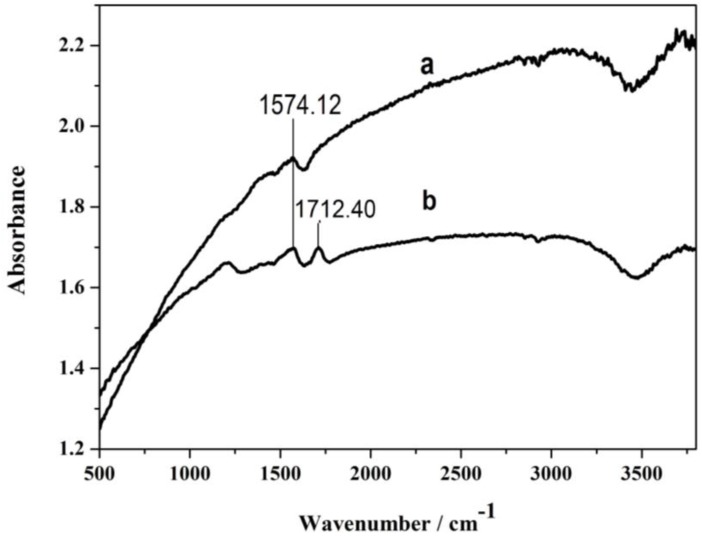
FT-IR spectra of MWCNTs (curve a) and carboxyl MWCNTs (curve b).

The Fourier transform infrared (FT-IR) spectra of multi-walled carbon nanotubes (curve a) and carboxyl multi-walled carbon nanotubes (curve b) are shown in Figure 5, where a characteristic carboxyl peak emerged at 1712.40 cm^−1^ (curve b), suggesting the carboxyl introduction into the multi-walled carbon nanotubes was successful. The electrical catalytic properties of bare multi-walled carbon nanotubes was improved. Simultaneously, there was carbon-carbon single bond vibration peak at 1574.12 cm^−1^ in curve a and curve b.

#### 4.1.3. Inverted Metallurgic Microscope Images of PME

**Figure 6:**
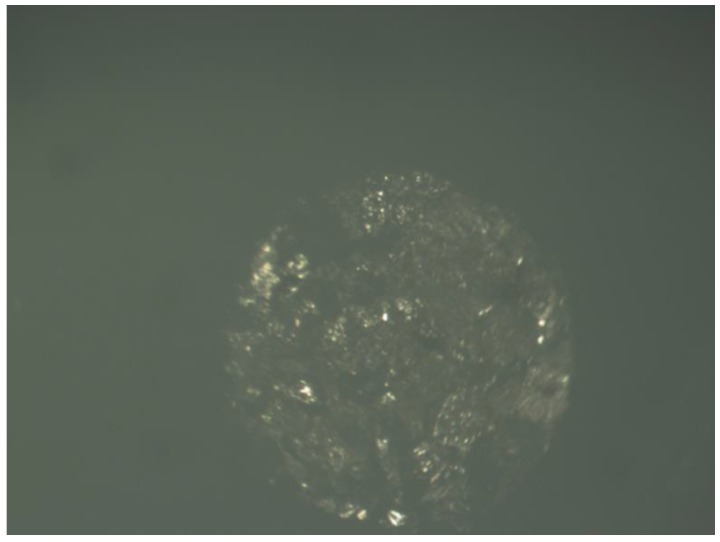
Inverted metallurgic microscope images of PME.

Images of embedded PME were obtained by an inverted metallurgic microscope (XB, 400XCE, Shanghai, China). According to Figure 6, the surface of platinum wire could be seen clearly. Before insertion, the tip of platinum wire had a bright facula, suggesting that there was nothing on the front of the platinum wire tip. After imbedding of carboxyl multi-walled carbon nanotubes, the facula wasn’t bright anymore and black powder was evenly distributed on the platinum wire tip, hinting that functional material was embedded on the platinum wire and the PME was fabricated successfully.

#### 4.1.4. Electrochemical Characterization of DMF/Carboxyl/MWCNTs/ME and PME

**Figure 7:**
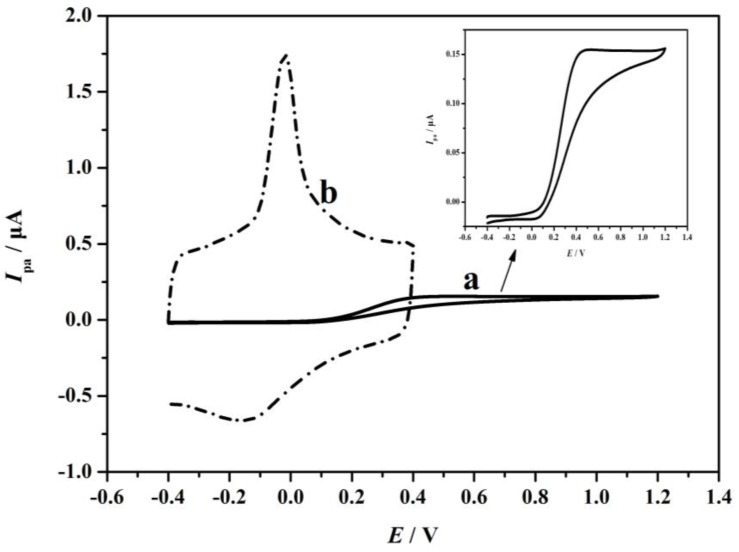
Cyclic voltammograms of 1.0 × 10^−2^ mol·L^−1^ AA (pH 6.0) on DMF/carboxyl/MWCNTs/ME (curve a) and PME (curve b) with scan rate of 0.10 V/s.

1.0 × 10^−2^ mol·L^−1^ AA solution (pH 6.0) was detected by cyclic voltammetry on a DMF/carboxyl/MWCNTs/ME (curve a) and PME (curve b), respectively. The results are displayed in Figure 7. The corresponding peak currents of curve a and curve b were 1.544 × 10^−7^ and 1.028 × 10^−6^ A. The peak potentials of curve a and curve b were 0.485 V and −0.020 V, respectively. Thereinto, the peak of AA on DMF/carboxyl/MWCNTs/ME was not obvious. When PME was used to detect AA, an apparent peak pattern was obtained. Compared to those of DMF/carboxyl/MWCNTs/ME, the AA’s peak current increased by 5.65 times and the peak potential was shifted to −0.020 V on the PME. According to the data, the carboxyl multi-walled carbon nanotubes introduced by embedding instead of chemical modification will enhance the electrocatalytic activity of materials and effectively improve the electrical activity of electrodes.

The active surface areas of DMF/carboxyl/MWCNTs/ME and PME were estimated according to the slope of *I_p_* versus *v*^1/2^ in a known concentration of K_4_Fe(CN)_6_, based on the Randles-Sevcik equation. The Randle-Sevcik equation is a linear scanning reversible wave equation under the condition of semi-infinite diffusion, which represents the relation between the current and the scanning speed of the electric potential. This equation is used to determine the diffusion coefficient or to determine the electrochemical area of an electrode:

*I_p_*= 2.69 × 10^5^*n*^3/2^*AD*^1/2^*v*^1/2^*C*_0_ (1)

where *I_p_* refers to the anodic peak current, *n* is the electron transfer number, *A* is the surface area of the electrodes, *D* refers to the diffusion coefficient, *C*_0_ is the concentration of K_4_Fe(CN)_6_, and *v* is the scan rate. According to the slope of *I_p_* versus *v*^1/2^, the microscopic areas were calculated to be 0.222 × 10^−4^ cm^2^ for DMF/carboxyl/MWCNTs/ME and 8.623 × 10^−3^ cm^2^ for PME. The results further show that the imbedding of functional powder would increase the specific surface area and the active sites of electrodes. It means that the process of treated ME becoming PME would improve the sensitivity of the electrode immensely.

### 4.2. Optimization Process

#### 4.2.1. Effect of Solution pH on AA Detecting 

**Figure 8:**
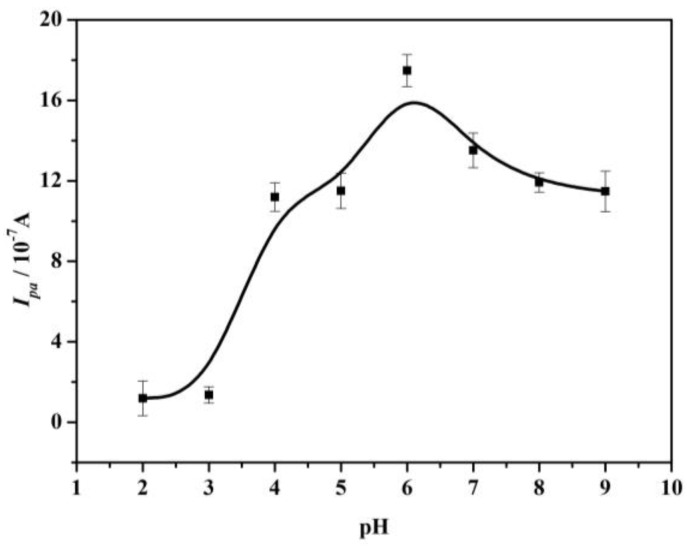
Influence of pH on the oxidation peak current of 1.0 × 10^−2^ mol·L^−1^ AA. Each point represents the average of three replicates and the standard deviation of the mean.

The influence of pH on the oxidation peak current of 1.0 × 10^−2^ mol·L^−1^ AA was investigated by cyclic voltammetry and the corresponding curve is shown in Figure 8. The AA oxidation peak current increased gradually from pH 1.0~6.0 and then decreased from pH 6.0~9.0. The maximum peak current was obtained at pH 6.0. When the pH of the solution was lower than 4.04 (pH < pK_a1_), AA was in the acidic form and the high concentrations of the acidic form will impact the reaction kinetics of AA with carboxyl multi-walled carbon nanotubes on the electrode surface. As the solution pH increased (pH > pK_a1_) [35,36], AA existed as a negatively charged species and the current of AA was increased. Thereinto, the effect of pH was the main factor in the activity of multi-walled carbon nanotubes. A current decrease was observed when the solution pH was higher than 6.0, due to the fact the electrode surface had more negative charge, hence the AA peak current was decreased. In conclusion, the pH of solution was chosen as 6.0 for the following tests to achieve a higher peak current.

#### 4.2.2. Effect of Sweep Rates on AA Detecting

**Figure 9:**
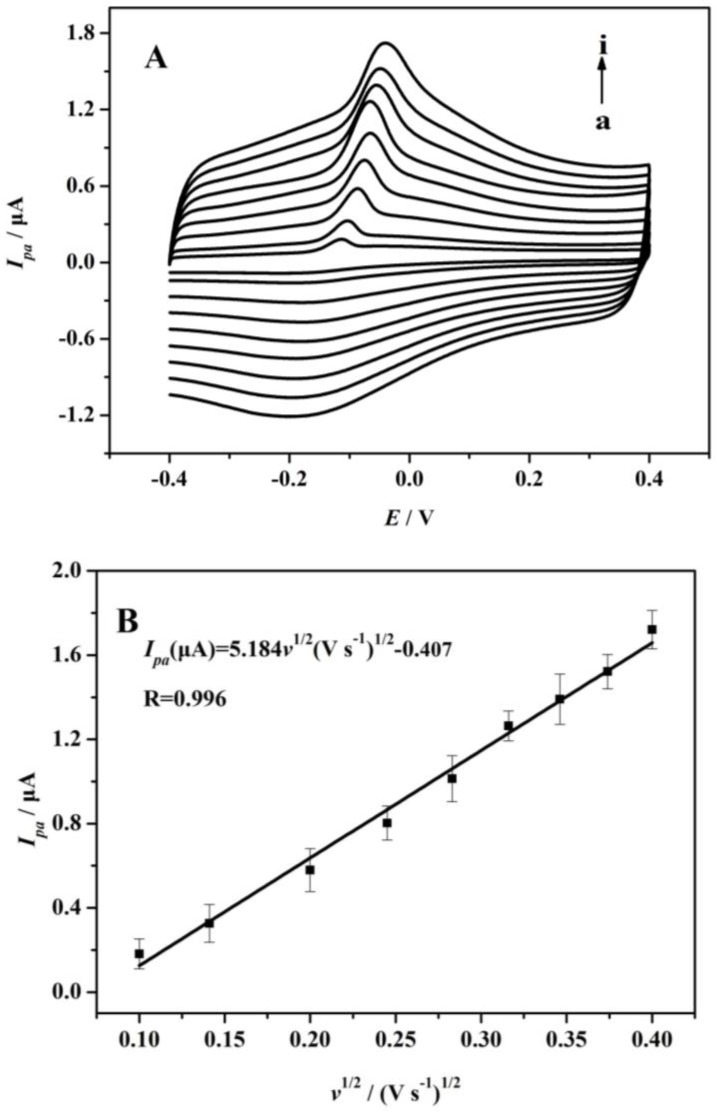
(**A**) Cyclic voltammograms of 1.0 × 10^−2^ mol·L^−1^ AA at PME with various sweep rates: (a–i) 0.01, 0.02, 0.04, 0.06, 0.08, 0.10, 0.12, 0.14, 0.16 V·s^−1^; (**B**) Plot of AA’s *I_pa_* versus *v*^1/2^. Each point represents the average of three replicates and the standard deviation of the mean.

The effect of sweep rates on the peak current of AA was also studied under optimum conditions (Figure 9).

It can be seen that the oxidation peak shifted to a more positive value for AA with increasing sweep rates along with a concurrent increase in current. The cyclic voltammogram data denoted that the anodic peak current of AA was increased linearly with the square roots, ranging from 0.10~0.16 V·s^−1^, implying that AA oxidation was controlled by a diffusion step, fitting the following equation [37,38]:

*I_pa_*(μA) = 5.184*v*^1/2^(V·s^−1^) − 0.407 (R^2^ = 0.991) (2)

The Cottrell equation is often used to determine the diffusion coefficient of solutions. The use of the Cottrell equation must satisfy the condition of semi-infinite diffusion. At the same time, any microelectrodes in the transient region follow the Cottrell equation. The dependence of peak potential (*E_pa_*) and ln(*v*) also showed a linear relationship with a regression equation of:

*E_pa_*(V) = 0.024ln(*v*)(V·s^−1^) − 0.002 (R^2^ = 0.976) (3)

According to the following equation [39]:

*E_pa_* = *E*°^/^ + m[0.78 + ln(*D*^1/2^*k_s_*^−1^) − 0.5ln*m*] + (*m*/2)ln(*v*) (4)

with:

*m* = *RT*/[(1 − α)*n_α_F*] (5)

where *E_pa_* is the oxidation peak potential of AA, *v* is the sweep rate, *E*°^/^ is the formal potential, *k_s_* is the electron transfer rate constant, and *n_α_* is the number of electrons involved in the rate determining step. The value of *m* = 0.048 was calculated from Equation (4) with *R* = 8.314 J·(mol·K)^−1^, *T* = 298 K and *F* = 96485 C·mol^−1^. Thus, the electron transfer coefficient (α) for AA oxidation on the PME was calculated to be approximately 0.73 by Equation (5) [38].

**Figure 10:**
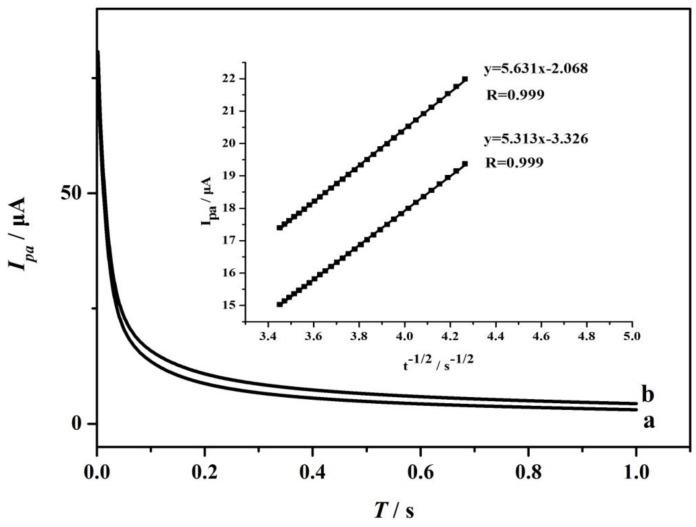
Chronoamperograms obtained at PME in the presence of 1.0 × 10^−3^ mol·L^−1^ (curve a) and 1.0 × 10^−2^ mol·L^−1^ (curve b) AA in the buffer solution (pH = 6). Inset: Cottrell’s plot for the data from the chronoamperograms.

The electro-oxidation for AA on PME was studied by potential chronoamperometry (Figure 10). The working electrode potential was set at 0.60 V to complete AA’s chronoamperometric measurements. Based on chronoamperometric method, AA’s diffusion coefficient (*D*) can be confirmed. The experimental plots of *I_pa_* versus *t*^−1/2^ were employed with the best fit for different concentrations of AA. The slopes of the resulting straight lines were plotted versus AA concentrations according to the Cottrell equation [40]:

*I* = *nFAD*^1/2^*C*/π^1/2^*t*^1/2^ (6)

The *D* was calculated as 7.634 × 10^−4^ cm^2^·s^−1^, which was larger than in previous reports [37,38,39,40,41,42,43,44,45,46,47,48,49,50].

### 4.3. Determination of AA

**Figure 11:**
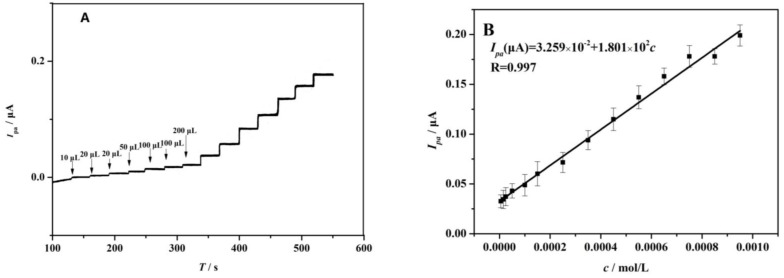
(**A**) Amperometric response curves of PME with successive additions of different concentrations; (**B**) Calibration curve of amperometric response *I_pa_* versus AA concentration. Each point represents the average of three replicates and the standard deviation of the mean.

Under the optimized experimental conditions, the PME was applied to detect a series of AA solutions by amperometry to investigate its sensitivity and detection performance. Figure 11 shows current-time plots of the PME with successive addition of AA. When a certain volume of AA was added to the buffer solution, the peak current showed a remarkable increase and quickly reached steady state. The concentrations of AA had a linear relation to the peak current of 5.00 × 10^−6^~9.50 × 10^−4^ mol·L^−1^: *I_pa_*(μA) = 3.259 × 10^−2^ + 1.801 × 10^2^*c*, R^2^ = 0.993. The detection limit (LOD) and quantitation limit (LOQ) were 4.89 × 10^−7^ mol·L^−1^ and 1.63 × 10^−7^ mol·L^−1^, respectively. From these data, it can be concluded that AA oxidation on the PME can be used to detect AA in a wide range of concentrations with high sensitivity. For the determination performance, PME’s LOD was the equivalent to or better than that of AA detection sensors in the literature, as shown in Table 1.

### 4.4. Interference Study

Various foreign species’ influence on the determination of 1.0 × 10^−2^ mol·L^−1^ AA was investigated. The potentially interfering substances were chosen from species commonly found with AA in biological fluids or pharmaceuticals. According to the experimental results, we can conclude that 200-fold excess of Mg^2+^, Ca^2+^, K^+^, Na^+^, Cl^−^, fructose, sucrose, 100-fold excess of glucose, glycine, valine, methionine, vitamin E, polyphenol, 30-fold excess of aspirin and 10-fold excess of dopamine have no apparent influence on AA detection. Simultaneously, the matrix effects of common matrices, which were all higher than 95%, are listed in Table 2.

### 4.5. Sample Analysis

To assess the PME’s practical application, 15 g samples (such as lemon, apple, vitamin C tablets) were chopped, ground and centrifuged at 6000 r/min for 10 min. The supernatant and AA standard liquid was added to the PBS buffer solution in turn by the standard addition method. The results are shown in Table 3, where it can be seen that satisfactory recoveries (94~107%) were obtained for PME using practical samples, suggesting the proposed method is effective and can be used to detect AA in practical samples.

### 4.6. Repeatability and Stability of the PME

A low relative standard deviation (RSD) of 2.068% was observed for measurements of 1.0 × 10^−2^ mol·L^−1^ AA activity with 12 replicate measurements, indicating excellent repeatability of the response of the PME. The longtime stability of the PME was also evaluated by determining 1.0 × 10^−2^ mol·L^−1^ AA. When not in use, the electrode was stored at 4 °C. The average value of the electrode response for the three measurements was gradually decreased to about 90% of the initial value over a period of 1 week.

## 5. Conclusions

In summary, carboxyl functionalized multi-walled carbon nanotubes were synthesized and embedded in a corroded electrode for highly sensitive electrochemical detection of AA. The carboxyl multi-walled carbon nanotubes showed an amplification effect on the electrochemical response, which was attributed to the carboxyl reactions of the multi-walled carbon nanotubes. More importantly, the functional nanomaterial was introduced by embedding instead of chemically modifying the ME. The PME possesses a large surface and this accelerates electron transfer, thus making the dual-electrode sensor exhibit significantly improved signal-acquisition rates. Practical sample analysis revealed the dual-electrode sensor system exhibited good accuracy, specificity and reproducibility. These features made this sensor system favorable for detecting AA at low concentration levels and shows promising applications in food analysis and clinical research.

## Figures and Tables

**Table 1:** Analytical characteristics for AA detecting of comparable methods.

Electrode	Method	Potential	Linearity Range	LOD	Real Sample	Reference
Tm_2_O_3_/ITO	CV	0.60 V	0.2~8 mmol·L^−1^	0.42 mmol·L^−1^	No mentioned	[19]
Pan/SWi_12_/TiO_2_-MoO_3_	DPV	0.27 V	0.95~6.9 mmol·L^−1^	1.2 μmol·L^−1^	Tomato, orange juice	[41]
PAn-β-naphthalenesulfonic acid	CV	0.35 V	5~60 mmol·L^−1^	12.93 μmol·L^−1^	Not mentioned	[42]
Molecularly imprinted PAN	DPV		0.05~0.4 mmol·L^−1^	18 μmol·L^−1^	Vitamin C tablet	[43]
An-β-aminobenzenesulfonic acid	DPV	0.15 V	35~175 μmol·L^−1^	7.5 μmol·L^−1^	Vitamin C tablet	[44]
Fc^+^-thioglycolate	DPV	0.164 V	1.0~500 μmol·L^−1^	0.2 μmol·L^−1^	Human urine	[45]
Poly-Trypan Blue	DPV	0.15 V	1.0~630 μmol·L^−1^	0.1 μmol·L^−1^	Vitamin C/serum	[46]
L-Cysteine sonogel-carbon	SWV	−0.1 V	0.05~1 mmol·L^−1^	0.05 mmol·L^−1^	Serum	[47]
CeO_2_ NP/GC	DPV	0.1 V	1.0~500 μmol·L^−1^	5 μmol·L^−1^	Serum	[48]
PdNi/C/GCE	Amperometric response		0.01~1.8 mmol·L^−1^	0.5 μmol·L^−1^	Vitamin C	[49]
PME	Amperometric, CV	−0.02 V	5.0~950 μmol·L^−1^	0.489 μmol·L^−1^	Lemon	This work

**Table 2:** Matrix effect studies for 1.0 × 10^−2^ mol/LAA.

Matrix	AA’s Peak Current Value before Matrix Addition (μA)	AA’s Peak Current Value after Matrix Addition (μA)	Matrix Effect
2 mol·L^−1^ KCl	8.563	8.493	99.18%
2 mol·L^−1^ NaCl	8.357	8.270	98.95%
2 mol·L^−1^ MgSO_4_	8.235	7.985	96.97%
2 mol·L^−1^ NH_4_Cl	8.727	8.431	96.61%
2 mol·L^−1^ CaCl_2_	8.128	7.786	95.79%
1 mol·L^−1^ glucose	8.176	8.085	98.89%
2 mol·L^−1^ sucrose	8.319	7.962	95.71%
2 mol·L^−1^ fructose	8.256	8.167	98.92%
1 mol·L^−1^ vitamin E	8.437	8.230	97.54%
1 mol·L^−1^ polyphenol	8.356	8.043	96.25%

**Table 3:** Recovery studies for AA in samples (n = 3).

Sample	Additive (10^−5^ mol·L^−1^)	Found (10^−5^ mol·L^−1^)	Recovery (%)	RSD (%)
Lemon	1.00	0.97	97%	4.7
	2.00	1.91	96%	3.9
	3.00	3.14	105	4.5
Apple	1.00	0.95	95%	3.3
	2.00	1.95	98%	2.9
	3.00	2.83	94%	2.5
Vitamin C tablets	1.00	0.96	96%	4.1
	2.00	2.14	107%	4.6
	3.00	2.92	97%	3.7
